# Thinking Outside the Box: Innate- and B Cell-Memory Responses as Novel Protective Mechanisms Against Tuberculosis

**DOI:** 10.3389/fimmu.2020.00226

**Published:** 2020-02-14

**Authors:** José Alberto Choreño-Parra, León Islas Weinstein, Edmond J. Yunis, Joaquín Zúñiga, Rogelio Hernández-Pando

**Affiliations:** ^1^Escuela Nacional de Ciencias Biológicas, Instituto Politécnico Nacional, Mexico City, Mexico; ^2^Laboratory of Immunobiology and Genetics, Instituto Nacional de Enfermedades Respiratorias Ismael Cosío Villegas, Mexico City, Mexico; ^3^Section of Experimental Pathology, Department of Pathology, Instituto Nacional de Ciencias Médicas y Nutrición Salvador Zubirán, Mexico City, Mexico; ^4^Department of Cancer Immunology and AIDS, Dana-Farber Cancer Institute, Boston, MA, United States; ^5^Department of Pathology, Harvard Medical School, Boston, MA, United States; ^6^Tecnologico de Monterrey, Escuela de Medicina y Ciencias de la Salud, Mexico City, Mexico

**Keywords:** *Mycobacterium tuberculosis*, trained immunity, B-cells, memory-like NK cells, ILCs

## Abstract

Tuberculosis (TB) is currently the deadliest infectious disease worldwide. Failure to create a highly effective vaccine has limited the control of the TB epidemic. Historically, the vaccine field has relied on the paradigm that IFN-γ-mediated CD4+ T cell memory responses are the principal correlate of protection in TB. Nonetheless, the demonstration that other cellular subsets offer protective memory responses against *Mycobacterium tuberculosis* (Mtb) is emerging. Among these are memory-like features of macrophages, myeloid cell precursors, natural killer (NK) cells, and innate lymphoid cells (ILCs). Additionally, the dynamics of B cell memory responses have been recently characterized at different stages of the clinical spectrum of Mtb infection, suggesting a role for B cells in human TB. A better understanding of the immune mechanisms underlying such responses is crucial to better comprehend protective immunity in TB. Furthermore, targeting immune compartments other than CD4+ T cells in TB vaccine strategies may benefit a significant proportion of patients co-infected with Mtb and the human immunodeficiency virus (HIV). Here, we summarize the memory responses of innate immune cells and B cells against Mtb and propose them as novel correlates of protection that could be harnessed in future vaccine development programs.

## Introduction

*Mycobacterium tuberculosis* (Mtb), the causative agent of pulmonary tuberculosis (TB), remains the most important pathogen worldwide in terms of accumulated mortality. The World Health Organization has estimated that 10 million new cases of TB and 1.421 million deaths caused by Mtb occurred in 2018 (1). The convergence of the Mtb and human immunodeficiency virus (HIV) epidemics, as well as the lack of new vaccines capable of conferring significant protection against TB have limited the control of this global health treat. Failure to create an effective vaccine for TB has been largely due to an incomplete understanding of the immune mechanisms associated with protective immunity against Mtb. In fact, for many years, the TB vaccine field has established the paradigm that CD4+ T memory cell responses mediated by IFN-γ are the chief immune mechanism which controls the spread of Mtb within the infected lung (2, 3). Despite its relevance, this mechanism has erroneously been considered the sole correlate of protection in TB (4). Moreover, recent findings have raised uncertainty about the protective capacity of IFN-γ-mediated CD4+ T cell memory against Mtb. For instance, T cell epitopes have been demonstrated to be well-conserved in Mtb, suggesting that the pathogen may take advantage of its recognition by T cells (5). Furthermore, recent TB vaccine candidates targeting IFN-γ-mediated T cell functions have failed to provide improved effectiveness compared to the *Mycobacterium bovis* Bacillus Calmette-Guerin (BCG) vaccine (6). Finally, IFN-γ has shown a poor predictive value in discriminating between subjects receiving BCG vaccination that will receive protection from those that will develop active TB (7). The discussion of the protective capacity of T cell memory responses against Mtb is beyond the scope of the present review, but further evidence has been extensively revised and analyzed by other researchers (8).

Hence, the TB vaccination field would benefit from the exploration of novel correlates of protection and the development of new strategies to disrupt the natural immune responses induced by Mtb to ensure its survival. Recently, some authors have proposed that this goal could be achieved through two complementary approaches: 1) inducing immune memory responses lacking or being strong enough to overcome the characteristics of the natural anti-Mtb immune responses that are beneficial for the pathogen, but with minimal risk of immunopathology, or 2) triggering very early protective responses that prevent the establishment of evasive mechanisms used by Mtb to manipulate the innate immune response (9). A growing body of evidence suggests that these approaches could be achieved by targeting immune cell populations other than T cells (10–13). In particular, it has been increasingly accepted that B cells actively participate in anti-Mtb immunity, either as secondary actors providing support and shaping the quality of T cell-memory responses, or as protagonists mediating direct effector functions against Mtb (14). Similarly, different subpopulations of innate immune cells that possess a previously unrecognized capacity to mount secondary “memory-like” responses are equally capable of limiting Mtb growth (11, 15). Therefore, in this review we summarize the memory responses of innate immune cells and B cells against Mtb and analyze how their functions may constitute novel correlates of protection that can be potentially harnessed for TB vaccine development.

## Memory Responses Against Mtb Within the Innate Immune System

As mentioned before, the study of the mechanisms underlying immunity to Mtb infection has focused on immunological memory mediated by adaptive immune cells, mainly CD4+ T helper lymphocytes. However, human studies have shown that up to a quarter of the individuals that are in close contact with active TB patients remain clear of the infection (16). These individuals test negatively in the purified protein derivative (PPD) skin test and IFN-γ release assays (IGRAs) (16, 17), which are two indirect readouts of adaptive responses against immune-dominant Mtb antigens. A positive BCG vaccination history has been associated with this state of immune protection (16). This suggests that in such “resistant” close-contacts, innate immune responses potentiated by BCG vaccination are sufficient to restraint Mtb infection.

Notably, recent investigations have uncovered the role of “trained immunity” in the pathogenesis of different infectious diseases. This concept describes an enhanced capacity of innate leukocytes to respond to secondary challenges by the same or by unrelated microorganisms after an initial antigenic exposure (18). The first hints of trained immunity induced by mycobacterial components were observed in mice treated with muramyl dipeptide (19), Freund's complete adjuvant (20), and BCG/PPD (21). Mycobacterial component preparations conferred protection against *Klebsiella pneumoniae*, foot and mouth disease virus (FMDV), and *Candida albicans* infection, respectively. Interestingly, protection from FMDV was independent of neutralizing antibodies (20), whereas control of systemic candidiasis occurred before the development of delayed-type hypersensitivity (21). Similarly, BCG exposure was shown to limit *Schistosoma mansoni* infestation in nude mice, suggesting a role for trained immunity in BCG-induced cross-protection (22). In humans, BCG vaccination conferred heterologous immunity against lower respiratory tract pathogens (23, 24), yellow fever virus (25), and neonatal sepsis (24). Furthermore, BCG vaccination was demonstrated to induce protection against intestinal parasites (26), even in individuals infected with HIV, suggesting that innate immune cells mediate such cross-protection.

Epigenetic changes that facilitate the expression of inflammatory genes and generate metabolic reprogramming are responsible for inducing trained immunity against mycobacterial and other microbial components. In these epigenetic changes, long non-coding RNAs participate in the formation of chromatin loops that bring several genes into a close spatial relationship, making these genetic regions more accessible to the enzymatic complexes responsible for epigenetic priming. Three cellular subsets appear to be involved in the trained immune response against Mtb: monocyte-macrophages, myeloid cell precursors, and innate lymphoid cells (ILCs), these latter including natural killer (NK) cells (18).

### Myeloid Cells in Memory Responses Against Mtb

Infected cell recognition is needed for the clearance of any infection. The adequate induction of protective immune responses against Mtb is initiated through efficient bacterial antigen recognition via pattern recognition receptors (PRRs) present in antigen presenting cells (APCs). Monocytes and macrophages are myeloid cells that participate in pathogen clearance at different organs. Irrespective of their origin, macrophages acquire a distinctive tissue-residency phenotype under the influence of local signals (27). In the lung, two subpopulations of macrophages maintain tissue homeostasis. Precursors of alveolar macrophages (AMs) from the yolk sac infiltrate the alveolar epithelium during fetal development, maintaining themselves through a self-renewal process independent of circulating monocytes (28). These phagocytes eliminate infectious agents that reach the lumen of distal airways, including Mtb (29). On the other hand, monocyte-derived macrophages migrate to the lung interstitium to participate in the elimination of pathogens, as well as in antigen presentation, and the recruitment of other leukocytes during inflammation (30).

AMs are among the first cells of the host immune system to encounter Mtb. After engulfing the bacillus, they leave the airways and initiate a cascade of inflammatory signals that will lead to the recruitment of more leukocytes around the sites of Mtb exposure (29). Over time, infiltrating leukocytes form structures called “granulomas” where different subtypes of phagocytes interact with the bacillus (31). Phagocytosis, antigenic processing, and cytokine production by myeloid cells shape the subsequent adaptive immune response (32). Nevertheless, Mtb employs mechanisms to evade the bactericidal activity of macrophages within immature phagosomes (i.e., Rab5 blockage, respiratory oxidative burst avoidance, etc.) establishing a proliferative niche (33). Hence, it was believed that pulmonary Mtb infection was contained only when primed antigen specific CD4+ T cells migrated to the lung parenchyma to stimulate antimicrobial mechanisms of local macrophages (34, 35). As a result, researchers have attributed excessive importance to the adaptive immune response in TB and far fewer significance to the trained immune response. The current view has probably limited the potential of basic and clinical research in TB vaccination strategies. Notwithstanding, this dogma is changing as emerging evidence demonstrates that the outcome of TB might be significantly influenced by special features present in innate immune cells, including their capacity to develop trained immunity. Moreover, the qualities that resemble memory in monocytes and macrophages are fueling the investigation of novel methods to potentiate innate defenses against Mtb (11).

Early studies in mice showed that BCG-induced trained immunity was mediated by macrophages (20, 21). Indeed, macrophages from animals treated with BCG produce more reactive oxygen species (ROS) and have increased *Candida albicans* growth restriction (21). In humans, BCG vaccination greatly enhances the ability of monocytes to produce inflammatory cytokines, such as IL-1β, TNF-α, and IFN-γ, after secondary encounters with Mtb and other microorganisms (36). Moreover, these monocytes expressed larger quantities of CD11b and toll-like receptor 4 (TLR4), two important pattern recognition receptors that initiate phagocytosis (37–39), suggesting that trained-monocytes may be more efficient at detecting and engulfing Mtb. The innate memory responses of monocytes depend on the intracellular recognition of BCG via NOD2 receptor and downstream Rip2 kinase signaling (36). NOD2 is one of the main receptors triggering immune activation after Mtb uptake (40) and is also involved in the initiation of autophagy (41). Thus, autophagy mediated by the NODS-Rip2 pathway is one of the mechanisms necessary for the development of trained immunity. Moreover, pharmacological inhibition of autophagy abolishes trained immunity in BCG-treated human monocytes (42). Interestingly, in some trials, the effect of BCG-induced training of human monocytes was still present 3 months after vaccination (36). This raised questions about the processes implicated in the maintenance of innate memory in monocytes because of the short half-life of these circulating cells (43).

Besides monocytes and macrophages, bone-marrow myeloid precursors were also reported to receive epigenetic reprogramming after microbiota exposure (44). Based on this finding, Kaufmann et al. exposed mice to intravenous BCG and observed an IFN-γ-dependent expansion of hematopoietic stem cells (HSCs) within the bone marrow (11). These HSCs displayed an increased expression of genes implicated in myeloid cell differentiation, inflammation, and IFN-γ signaling responses. Furthermore, bone marrow-derived macrophages (BMDMs) from mice exposed to intravenous BCG possessed an increased capacity to restrict Mtb growth *in vitro*, and conferred protection from aerosol Mtb infection when adoptively transferred into Rag1 immunodeficient mice (11). Such protection was independent of antigenic persistence in donor mice exposed to intravenous BCG, as antibiotic administration to donor mice before adoptive transfer did not influence the capacity of BMDMs to confer protection against Mtb *in vivo*. Interestingly, the epigenetic changes associated with immune training of BMDMs were similar to those observed in human innate immune cells trained by BCG vaccination (36), as well as by exposure to other microbial components (45). These epigenetic modifications consisted of increased H3K4me3 histone marks at promoters of genes involved in inflammation and anti-Mtb responses (11, 36). Accordingly, pharmacologic inhibition of histone methylation reversed BCG-induced training of human monocytes (36). Thus, trained monocytes/macrophages mount enhanced secondary responses to Mtb because they carry epigenetic modifications that make inflammatory genes more accessible to the transcriptional machinery. In addition, BCG-induced histone modifications in trained-myeloid cells cause a metabolic shift in these cells which potentiates glycolysis and glutamine consumption pathways (46). Hence, the improved effector capacity of BCG-trained macrophages is related to their increased catabolic activity and energetic production required to sustain their pro-inflammatory and bactericidal actions.

**Figure 1:**
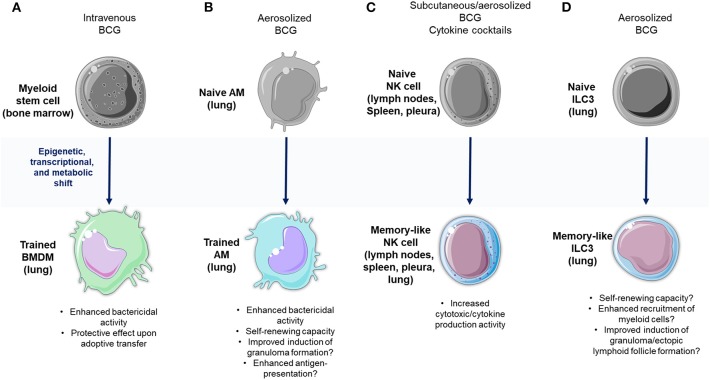
Memory-like responses against Mtb within the innate immune system**. (A)** Intravenous Bacillus Calmette-Guerin (BCG) vaccination induces the expansion of human stem cells (HSCs) and a bias toward the production of myeloid precursors carrying epigenetic imprints that reprogram their transcriptional and metabolic profiles. These cells subsequently generate a progeny of myeloid cells with enhanced effector functions that confer protection against aerosol infection in Mtb-infected mice. **(B)** Alveolar macrophages (AMs) display characteristics of trained immunity, which may provide protection against Mtb infection. **(C)** BCG as well as natural Mtb infection potentiate the effector functions of natural killer (NK) cells, and induce the expansion of memory-like NK cell subpopulations. **(D)** Similarly, innate lymphoid cells (ILCs) possess characteristics of adaptive immunity that may confer protection in TB, thus placing these cells as potential targets for vaccine development programs. The art pieces used in this figure were modified from Servier Medical Art by Servier, licensed under a Creative Commons Attribution 3.0 Unported License (https://smart.servier.com/).

These findings open the possibility for evaluating novel vaccination strategies targeting the bone marrow (Figure 1A). However, it is crucial to confirm the occurrence of epigenetic priming of myeloid precursors after BCG administration or during natural pulmonary TB infection in humans. Equally important is determining whether innate immune memory responses of HSCs and their progeny can generate significant adverse effects in the host. In fact, there is evidence showing that systemic inflammatory stimuli induce training of monocytes/macrophages which then mediate local tissue damage at different anatomical sites (47–49). Conversely, a recent study in macaques receiving intravenous BCG demonstrated that this route of vaccination, which perhaps reaches the bone marrow, is effective at conferring protection against Mtb infection without generating pathological effects (50). However, in this study, the participation of trained myeloid cell precursors in protective immunity against TB was not addressed. Training of tissue-resident macrophages located solely within the lung may constitute a more prudent strategy to improve innate immunity at the sites of natural Mtb exposure. In this context, recent findings provide evidence that AMs can also display memory characteristics in mice (51). The local priming of memory AMs has been triggered by mucosal infection of mice with an adenoviral vaccine vector, which caused an increased expression of MHC-II molecules and a metabolic shift toward glycolysis in these cells. Trained AMs protected animals against secondary infection with *Streptococcus pneumoniae*, maintaining their memory responses through a process of self-renewing which was independent of circulating monocytes. Hence, these data demonstrate that induction of trained immunity in lung AMs can be achieved by local antigen delivery and may explain why mucosal BCG vaccination is a better approach to confer protection against Mtb infection as compared to subcutaneous BCG administration (52–54). However, direct evidence of trained immunity induced by BCG in AMs is currently inexistent. Furthermore, although trained AMs contribute to the clearance of other respiratory pathogens (51), recent findings suggest that AMs are indeed more permissive than blood-derived monocytes to sustain the intracellular growth of Mtb (55). This contrasts with other data showing that CCR2+ AMs induce protective responses against Mtb (29). Therefore, it is likely that different subpopulations of AMs with variable bactericidal capacity against Mtb exist, mirroring M1 and M2 macrophages. Future studies are warranted to define whether memory AMs generate enhanced protection after mucosal BCG vaccination, as well as to determine the duration of their trained responses (Figure 1B). In addition, the pathogenic potential of trained AMs during TB must be addressed. If trained immunity is documented as a protective long-lasting response with limited adverse effects in TB, then it could emerge as a novel correlate of protection suitable to be targeted in vaccine development programs.

### NK Cell Memory in TB

NK cells participate in the defense against pathogens by mediating cytotoxicity against infected cells and through the production of a wide range of cytokines that influence the effector functions of other leukocytes (56). Activation of NK cells can be triggered by ligand recognition of infected cells via their activating receptors and through PRR microbial component recognition (57, 58). Furthermore, NK cells possess various inhibitory receptors that bind to MHC-I molecules and other related surface proteins (59). Cells disturbed by an intracellular infection lose the expression of their inhibitory ligands, allowing NK cells to react to the “missing self” signals by activating their effector functions (60).

NK cells are an important source of cytokines, chemokines and growth factors crucial for anti-Mtb immunity. In fact, they produce high amounts of IFN-γ (61), TNF-α (62), IL-17 (63), IL-22 (64, 65), and GM-CSF (66), all of which participate in TB pathogenesis (67). Thus, these cells have attracted the attention of researchers as they may play a relevant role in pulmonary TB. In this regard, it is known that NK cells can recognize several components of the Mtb cell wall through their activating receptors and PRRs (58, 68, 69). These interactions result in the production of proinflammatory cytokines that can increase the bactericidal activity of macrophages (70–72), as well as cytotoxic proteins that can kill bacilli-loaded phagocytes (73). *In vitro* assays have demonstrated that NK cells can even eliminate extracellular Mtb through cytotoxic mechanisms (74), although the relevance of this function has not been confirmed *in vivo*. In animal models, NK cells play a relevant role for protective immunity against Mtb only when T and B-cell responses are compromised (75). NK cells-derived IFN-γ and other proinflammatory cytokines (75, 76), potentiate the control of Mtb infection by macrophages. These functions are dependent on IL-12 and IL-21 production by T cells (75, 76). However, in immunocompetent animals NK cells play a minimal role in protective immunity against Mtb (77). In humans, NK cells can be found in lung tissue specimens from patients with chronic Mtb infection (78), suggesting their involvement in TB pathogenesis. Current evidence suggests a beneficial role of NK cells during human TB as changes in their immunophenotype and function are associated with the development of active TB (79–88). NK cells support the activity of CD8+ T cells during Mtb infection (89), shape the maturation process and Mtb-antigen processing in dendritic cells (DCs) (90), limit the expansion of regulatory CD25+ T cells (91), and even participate in the mechanisms underlying protection induced by BCG vaccination (92).

In a similar fashion to monocytes and macrophages, NK cells display characteristics of adaptive immune cells that allow them to mount recall responses against haptens, viruses, and bacteria (93–95). However, NK cell memory differs from trained immunity in myeloid cells in terms of the response specificity. In some cases, NK cell memory responses against specific antigens are mediated by a single subset of NK cells expressing a particular receptor (93, 94, 96). For instance, memory-like NK cells expressing CD94/NKG2C expand rapidly after cytomegalovirus (CMV) infection in humans (96). On the other hand, cytokine priming of NK cells can induce the expansion and development of memory responses against antigenic and non-antigenic stimuli (97, 98). Importantly, NK cell memory can be induced after BCG vaccination and after natural Mtb exposure in both mice and humans (99). For instance, NK cells isolated from BCG-vaccinated humans show an increased capacity of proinflammatory cytokine production after *ex vivo* re-challenge with Mtb, BCG, and other bacteria and fungi (99). Such enhanced responses are long-lasting and can be observed 1 year after BCG revaccination in LTBI patients (100). Human NK cells can also be primed during natural pulmonary Mtb infection in humans, as suggested by the expression of CD45RO, a molecular marker classically used to identify memory T cells subsets (101, 102). These human CD45RO+ NK cells demonstrate increased cytotoxicity and IFN-γ production capacity after *ex vivo* stimulation with IL-12, as compared to CD45RO- NK cells (102). Additionally, CD45RO+ NK cells produce more IL-22 in response to IL-15 and BCG (101). However, the relevance of such trained-NK cell responses in protective immunity against Mtb infection in humans remains unclear.

In a recent study, a protective effect of memory-like NK cells against murine pulmonary TB was observed (15). BCG-vaccinated mice showed an IL-21 dependent expansion of NKp46+CD27+KLRG1+ NK cells in the lymph nodes and spleen. Such cells produced more IFN-γ than their NKp46+CD27− counterparts and were able to improve Mtb infection control both in isolated macrophages as well as in mouse TB-infected lungs after their adoptive transfer. The specificity of BCG-induced memory-like responses mediated by NK cells seemed to be limited to Mtb, as these cells did not respond to *Candida albicans*. In addition, higher amounts of CD56+CD27+ NK cells were observed in humans with LTBI compared to healthy donors, providing evidence of memory-like NK cell expansion in humans with controlled TB. Interestingly, human CD56+CD27+ NK cells have a higher capacity of limiting intracellular Mtb growth in macrophages compared to CD56+CD27-NK cells. Nevertheless, the protective role of memory-like NK cells in human TB remains unknown as this NK cell subpopulation was only evaluated in individuals with LTBI, but not in patients with active TB (15). In summary, memory-like NK cells promise to be new targets for TB vaccine development due to their possible protective functions (Figure 1C). Nonetheless, uncovering their precise role in human anti-Mtb immunity remains a challenge. Collectively, the evidence suggests that despite the apparent redundancy of NK cell functions during Mtb infection in immunocompetent hosts (77), the potentiation of their memory properties may be beneficial in subjects with impaired adaptive responses (75), such as those infected with HIV.

### Memory of ILCs in TB

ILCs are lymphocytes that functionally mirror adaptive T helper cells except for their lack of rearranged antigen-specific receptor expression. Hence, these cells are considered as part of the innate branch of the immune system. Type 1 ILCs are the innate counterpart of Th1 lymphocytes and, as such, produce high amounts of IFN-γ upon stimulation. ILC2s resemble Th2 cells, produce IL-4, IL-5, and IL-13, and play a role in allergic disorders and defense against parasites. ILC3s functionally mirror Th17 cells and produce IL-17 and IL-22 (103). Recently, ILCs have been implicated in the immune response to Mtb. Compared to healthy controls, all ILC subsets are diminished in the peripheral blood of humans with active TB. The proportion of circulating ILC1s and ILC3s is restored after infection clearance following antibiotic treatment (12). The depletion of circulating ILCs has been linked to their migration toward sites of Mtb exposure within the lung. In fact, during advanced pulmonary TB, ILCs are enriched in both mice and human lung tissue. Their recruitment is apparently regulated via the CXCL13/CXCR5 axis. Additionally, in humans, ILC2s and ILC3s localize within the infected lung and overexpress genes involved in inflammation and myeloid cell chemotaxis, such as CXCL17 (104). In mice, the maximum migration of ILCs to the lungs coincides with the peak recruitment of AMs. Absence of ILCs, particularly of ILC3s, results in a reduction of lung AMs recruitment, higher bacterial burden, and altered tertiary lymphoid nodule (TLN) and granuloma formation during TB infection (12). Similar findings were reported in type 2 diabetes mellitus (T2DM) mice infected with Mtb, in which the adoptive transfer of ILC3s prolonged their survival, limiting neutrophil accumulation within the lung, and preventing damage to the alveolar epithelium via the production of IL-22 (105). These findings suggest that ILCs, specifically ILC3s, participate in the immune response to Mtb. Nonetheless, additional TB models in animals with specific deletion of ILC3s are required to confirm these data.

Interestingly, recent investigations have uncovered the ability of ILCs to display features that resemble the trained immune responses of macrophages and the memory-like responses of NK cells (106). These innate memory-like responses of ILCs could be targeted via vaccination (Figure 1D). For instance, BCG vaccination in mice has already shown to induce rapid accumulation of ILCs within the lungs (107). In such animals, the intranasal route of BCG administration generated a stronger recruitment of lung ILCs which possess an enhanced ability to produce IFN-γ compared to the intradermal route of BCG administration. These data suggest that mucosal BCG vaccination may trigger the development of lung memory-like ILCs. Nevertheless, further experimental demonstration of the adaptive characteristics of such cells and their protective or pathogenic nature in the context of TB is required.

## B Cells in Memory Responses Against Mtb

The role of B cells in the defense against pathogens greatly relies on their capacity to generate immune memory, providing the host with a durable reservoir of antigen-specific antibodies, as well as a pool of long-lasting antibody-producing cells that can re-expand in case of a secondary challenge. During a primary response to an antigen, naïve B cells may transform into effector B cells and subsequently experience class switching, recombination, and affinity maturation within germinal centers (GC), with the support of follicular T helper (Tfh) cells. This GC reaction is a common pathway that gives rise to memory B cells (MBCs) and plasmablasts. MBCs secrete null amounts of antibodies but in case of a re-encounter with the same pathogen, they can generate plasmablasts within hours. Plasmablasts are B cells capable of secreting reduced amounts of antibodies and more importantly, are precursors of plasma cells (PCs), short-lived cellular units that use their protein machinery for the production and secretion of massive amounts of antigen-specific antibodies. PCs are unable to proliferate without antigenic stimulation; however, a selected minority of PCs can generate anti-apoptotic processes and migrate to the bone marrow, thus becoming long-lived PCs (LLPCs, also termed memory PCs). These LLPCs survive without antigenic stimulation and continuously liberate antigen-specific antibodies to the circulation (108, 109).

For many years, the role of antibody-mediated B cell memory responses in TB has remained controversial (110). However, several studies have demonstrated the importance of this response in combatting this disease. In fact, elevated serum titers of PPD-specific antibodies correlate with protection against TB in high-risk individuals and are a better indicator of LTBI than the skin test reaction (111). Such antibodies induce the stimulation of PBMCs *in vitro*, suggesting that after biding to its specific antigen, these immunoglobulins can trigger effector responses of different immune cells. In line with these findings, it has been recently described that the antibodies from LTBI subjects have an increased capacity to bind to the FcγRIII and trigger the effector activities of NK cells and macrophages (10). This functional difference is associated with distinctive patterns of glycosylation of the Fab domain of antibodies from LTBI patients compared to individuals with active TB (10). Functional humoral responses have also been linked to the development of sterilizing immunity in individuals resistant to TB. In such resistant subjects an IFNγ-independent response mediated by CD4+CD40L/CD154+ T cells induces T-follicular B cell help and the production of several non-Th subset-specific cytokines critical for B cell activation (112). Furthermore, in a recent study, the humoral response of this human population was characterized by an IgG1-dominant state specific to ESAT6/ CFP10, LAM and PPD. Conversely LTBI individuals displayed a diversified IgG subclass response (112). Additionally, antibodies from individuals with LTBI can restrict Mtb intracellular growth in a more successful fashion compared with antibodies from patients with active TB (113). Whether antibodies contribute to the “resistant” phenotype observed in some individuals remains an undetermined matter at this moment, however it is currently known that post-vaccination serum favors Mtb phagocytosis by macrophages, enhances phagolysosome fusion and inhibits intracellular Mtb growth (113). Furthermore, pooled IgG from LTBI individuals may eradicate intracellular bacilli through a process that likely involves the inflammasome (114). These findings suggest that antibody-mediated B-cell memory responses play a role in the defense against Mtb infection and may be targeted to induce protective immunity through vaccination. Unfortunately, the dynamics of the antibody mediated immune responses (AMIR) during TB progression remain insufficiently characterized. Nonetheless, as MBCs are not only located in lymphoid organs but can also be found in peripheral blood, some studies have examined the kinetics of circulating MBC populations in patients with distinct forms of TB.

**Figure 2:**
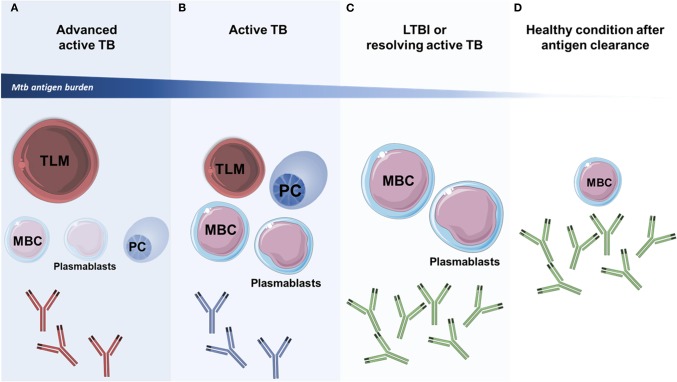
Profiles of circulating memory B cell subpopulations during TB. The characterization of the profile of circulating B cell subsets may help to discriminate different forms of the clinical spectrum of TB. **(A,B)** Most memory B-cells (MBCs), plasma cells (PCs), and plasmablasts subsets expand during active TB and depend on antigenic persistence. These subsets are depleted from the circulation of individuals with advanced TB, which show increased numbers of tissue-like memory B cells (TLMs) that have an exhaustion phenotype and are associated with disease progression. The humoral response in patients with active TB is characterized by the presence of antibodies that induce poor activation of effector activities in innate immune cells (antibodies in red and blue color). **(C)** Individuals with LTBI and those with resolving active TB are characterized by a strong functional antibody response (antibodies in green color), as well as by elevated numbers of circulating MBCs and plasmablasts. **(D)** In healthy individuals that are able to clear the infection, and in those that received antibiotic treatment, an increased proportion of circulating MBCs is observed. This cellular population may decrease over time in a parallel fashion to the antigenic burden. In these patients, a persistent functional antibody-mediated immune response is also observed (antibodies in green color). The size of B cell subsets at each specific stage of TB disease indicate their predominance (larger drawings) or downregulation (smaller drawings). The art pieces used in this figure were modified from Servier Medical Art by Servier, licensed under a Creative Commons Attribution 3.0 Unported License (https://smart.servier.com/).

Some preliminary findings suggest that the proportion of distinct MBC subsets may predict the clinical status of Mtb-infected patients (Figure 2), arguing in favor of a role of B cell memory in TB (108, 115–119). For instance, MBC, plasmablasts, and PCs subsets are significantly enriched in the circulation of patients with active TB, and their proportions diminish after the conclusion of anti-TB treatment, suggesting that these B cells subsets undergo a contraction phase after antigen clearance (119). Indeed, the maintenance of MBCs in circulation is dependent on the presence of Mtb antigens and can be eliminated after 12 weeks of antibiotic treatment (116), but serum IgG levels remain augmented even after a 6 month course of antibiotic therapy in TB patients (119). Thus, during active TB, the presence of the pathogen may trigger the generation of high-affinity antibody-mediated memory responses to Mtb antigens. Plasmablasts can readily induce class-switched and cytokine-producing PCs upon antigen exposure or re-exposure if they are derived from naïve B cells or antigen-experienced MBCs, respectively (14, 119). These responses may reduce Mtb burden in active TB patients and curb their disease outcome, as well as maintain a LTBI status. In fact, it was demonstrated that PCs derived from peripheral blood cells of LTBI subjects produce significant amounts of antibodies and IL-17 when exposed to Mtb antigens (115). Thus, the dual presence of MBCs and plasmablasts in the peripheral blood has been proposed as a marker of resolving active disease and LTBI. On the other hand, the presence of activated antigen-specific plasmablasts but not MBCs in the peripheral circulation may reflect the initial stages of active TB and the lack of both MBCs and plasmablasts in circulation may indicate uncontrolled infection (118). Particularly, non-switched IgD+ MBCs have been found reduced in distinct tissue compartments during advanced active TB (120, 121). Other studies have found that higher proportions of circulating MBCs but not plasmablasts in peripheral blood along with a prominent serum antibody-mediated memory response is indicative of a healthy condition after sterilizing immunity following Mtb infection (118). This coincides with the observation that healthy subjects who have resided in TB-endemic areas have greater frequencies of peripheral blood MBCs and antibody-mediated responses compared to subjects from other world areas (122). Therefore, although MBCs might contract after Mtb control, they remain preconditioned to mount secondary responses in case of reinfection.

The role and dynamics of LLPCs and marginal zone (MZ) B cells during TB is still under investigation. Recently, it was observed that BCG vaccination can elicit the generation of PPD-specific LLPCs in humans (122). Additionally, another study found that LLPCs are important contributors of cytokine production during LTBI (115). MBCs and LLPCs generate the AMIR through the production of IL-21 and the subsequent induction of a B cell maturation loop involving the formation of PCs (108, 116). Preliminary evidence suggests that this response is crucial for controlling Mtb infection (108, 116). The role of MZ B cells during TB has not been adequately characterized and inconsistencies have arisen between mouse and human studies (121). These cells are normally activated by T cell independent antigens and have innate-like B cell memory characteristics. MZ B cells contribute to the AMIR through the production of IgM and IgG3 antibodies (117). Interestingly, a pilot study indicated that MZ B cells are present in the peripheral circulation during active TB at significantly lower levels compared to healthy controls (116).

**Figure 3:**
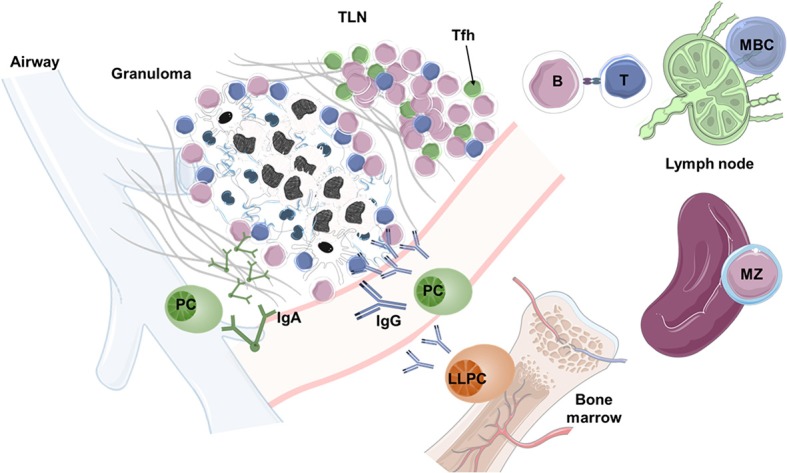
B cell functions in TB. During pulmonary Mtb infection, granulomas and peripheral tertiary lymphoid nodes (TLNs), which resemble germinal centers (GCs), develop in the lungs and shape the protective immune response. Within TLNs and local lymph nodes, B cells interact with Tfh cells, mediate antigen presentation, and produce soluble mediators that support the development of T cell memory responses. However, the identity of B cells that are present in TLNs needs detail characterization. In the bone marrow, strong antibody responses are generated by long-lived plasma cells (LLPCs) and circulating plasma cells (PCs), which are derived from memory B cells (MBCs). The antibody response generated by PCs may help to neutralize Mtb in both the mucosa (via IgA) and the peripheral circulation (via IgG). In case of reinfection, MBCs convert into plasmablasts and PCs, mounting a secondary antibody response within days. The art pieces used in this figure were modified from Servier Medical Art by Servier, licensed under a Creative Commons Attribution 3.0 Unported License (https://smart.servier.com/).

Beyond antibody production, B cells play a crucial role in regulating innate and adaptive immune responses to infectious agents even in disease states dominated by T lymphocytes, as is the case of TB. B cells impact cellular adaptive immunity and are necessary for the adequate activation and maturation of memory T cells (123–125). This regulation is achieved through direct interactions between B and T cells that occur during both primary and secondary antigen responses. Such interactions are varied and include antigen presentation, co-stimulatory signaling, cytokine priming and antibody-mediated effector functions, as shown in Figure 3. In fact, B cell-deficient mice and human patients receiving B cell depletion therapy generally present alterations in their CD4+ T cell and CD8+ T cell repertoires (126). However, B cells exert contrasting effects on T cell responses. For instance, B1 and B2 cell subsets are capable of producing IFN-γ, TNF-α, IL-12 or IL-2, IL-13, and IL-4, thus inducing Th1 or Th2 immune responses, respectively. Furthermore, B-cells also display different regulatory phenotypes capable of generating Th10 responses via IL-10, TGF-β, IL-35, FasL, or PD-L1. Besides T cell activation, B cells also regulate T cell proliferation and contraction during acute immune responses and participate in the maintenance and reactivation of T cell memory responses via antibody-independent mechanisms (127). Most of these B cell functions have not been adequately characterized during TB, although it is currently known that B cells can get infected by Mtb via micropinocytosis (128), and B cells can produce cytokines in response to infection with Mtb *in vivo* (129).

In addition, B cells are also crucial for the induction and maturation of antigen-presenting environments (APEs) and APCs (127). In TB, B cells participate in the formation of granulomas and play a pivotal role in the development of TLNs (Figure 3), two APEs that are critical for Mtb control (123, 124). As such, B cell-deficient mice infected with Mtb display disrupted lung granuloma and TLN formation (34). Moreover, the production of cytokines and antibodies by B cells contributes to set up activation thresholds for macrophages, DCs and other non-professional APCs within and outside APEs during TB (123), which subsequently will determine the quality and dynamics of effector and memory T cell responses. In this regard, during active pulmonary TB, humans, mice, and non-human primates develop TLNs in the surroundings of granulomas. These TLNs assume the structure and function of GCs containing B cells with different maturation profiles, Tfh-like CXCR5+ cells, and follicular DCs (34, 130). Additionally, pulmonary TLNs facilitate the interaction between these and other immune cells and become the anatomical foci that possess the highest levels of cellular proliferation during active TB. The presence and organization of these TLNs is associated with immune protection and LTBI, and their absence or disorganization may lead to uncontrolled progression of active TB (34).

Importantly, the possibility that Mtb might manipulate B cell and B cell memory responses exists, as B cells are susceptible to multiple effects of the bacteria including their direct infection via micropinocytosis (128). For instance, tissue-like memory B-cells (TLMs) are increased in the blood of patients with active TB and reduced in healthy controls (Figure 2). TLMs constitute a population of MBCs, also known as exhausted B-cells, that express low levels of CD21 and CD27 and increased levels of inhibitory receptors, having a diminished capacity to respond to antigenic stimuli. The presence of these cells in the circulation is associated with adverse clinical outcomes in chronic viral infections and is likely associated with active TB as well (128). Additionally, a population of B CD27-/IgD- memory cells has been shown to be increased in the pleural fluid of patients with tuberculous pleurisy as compared to healthy subjects. In these patients, pleural B cells interfered with the protective production of IFN-γ by NK cells and T lymphocytes (121). Collectively, these data indicate that targeting B cell functions for vaccination purposes in TB must be conducted carefully to avoid possible detrimental effects to the host.

## Conclusions and Future Directions

Humoral responses characterized by the production of high-affinity neutralizing antibodies have been the main target for the development of vaccines that confer protective immunity against several bacterial pathogens (108). Currently, the exception to the rule seems to be TB, as most of the TB vaccine candidates that are being evaluated in clinical trials are designed to exploit cell-mediated immunity rather than humoral immunity (131). Despite this, the evidence reviewed here suggests that both arms of the adaptive immune response should be evaluated in vaccine-development programs. Induction strategies should attempt to provide the host with a pool of antigen-specific MBCs capable of producing high-affinity neutralizing and polyfunctional antibodies which can potentiate macrophage and NK cell effector functions. Antibody-mediated activation of innate immunity may contribute to prevent the establishment of an immune microenvironment manipulated by Mtb. Vaccines targeting B cell responses can also promote the cooperation between B and T cells necessary for efficient T cell memory responses. For example, in a preclinical mouse model, the adoptive transfer of B cells that had internalized the naked plasmid pcDNA3 encoding the *Mycobacterium leprae* 65-kDa heat shock protein into B cell deficient mice resulted in the establishment of strong CD8+ T cell memory responses that conferred protection against Mtb challenge (132). Furthermore, novel ways to promote the formation of protective TLNs within the lungs deserves further investigation. Finally, the fact that a robust MBC response can be induced in the adjacent mesothelium upon primary mucosal Mtb exposure advocates in favor of using novel routes of vaccine administration (119, 121).

On the other hand, the memory-like properties of innate immune cells must also prompt us to discover novel ways to potentiate protective long-lasting anti-TB innate immune mechanisms. These strategies must aim to provide the host with a pool of primed long-lived innate cells with enhanced capacity to directly respond to the invading pathogen and with an increased adjuvant activity to support the correct development of effective T-cell memory responses. For instance, targeting trained immunity in myeloid cells may provide the host with metabolically and epigenetically reprogramed phagocytes ready to act before the contact with Mtb. Furthermore, memory-like AMs could initiate the early formation of protective granulomas around the sites of Mtb exposure, while the enhancement of ILCs activity could promote the establishment of an early immunological microenvironment advantageous for the host. Finally, Mtb-induced memory-like NK cells may serve as innate effector cells with increased cytokine production and cytotoxic activity. Targeting innate immunity in TB vaccination strategies may benefit a significant proportion of patients co-infected with Mtb and HIV by conferring them with T-cell independent protective immunity. HIV/AIDS remains the leading comorbidity in TB, and according to the 2019 WHO TB report, a third of HIV-related deaths were due to TB (1).

In conclusion, we must think outside the box and look for novel protective immune responses against Mtb beyond T cell memory responses. Future investigations in the TB field warrant the study of mechanisms implicated in trained immunity and B cell memory, as well as the discovery of routes and adjuvants for TB vaccine administration that may potentiate these and other overlooked protective immune responses against Mtb infection (131, 133).

## Author Contributions

JC-P and LW designed the study and drafted the manuscript. EY, JZ, and RH-P contributed in the writing process of the manuscript and revised it for intellectual content. All the authors revised and approved the final version of the manuscript.

### Conflict of Interest

The authors declare that the research was conducted in the absence of any commercial or financial relationships that could be construed as a potential conflict of interest.

## References

[1] World Health Organization WHO global tuberculosis report 2019. World Health Organization (2019). Available online at: https://apps.who.int/iris/bitstream/handle/10665/329368/9789241565714-eng.pdf?ua=1 (accessed on December 31, 2019).

[2] Orme IM Roberts AD Griffin JP Abrams JS. Cytokine secretion by CD4 T lymphocytes acquired in response to *Mycobacterium tuberculosis* infection. J Immunol. (1993) 151:518–25. 8100846

[3] Shimokata K Kishimoto H Takagi E Tsunekawa H. Determination of the T-cell subset producing gamma-interferon in tuberculous pleural effusion. Microbiol Immunol. (1986) 30:353–61. 10.1111/j.1348-0421.1986.tb00952.x 3088399

[4] Nunes-Alves C Booty MG Carpenter SM Jayaraman P Rothchild AC Behar SM. In search of a new paradigm for protective immunity to TB. Nat Rev Microbiol. (2014) 12:289–99. 10.1038/nrmicro3230 24590243 PMC4085047

[5] Comas I Chakravartti J Small PM Galagan J Niemann S Kremer K. Human T cell epitopes of *Mycobacterium tuberculosis* are evolutionarily hyperconserved. Nat Genet. (2010) 42:498–503. 10.1038/ng.590 20495566 PMC2883744

[6] Tameris MD Hatherill M Landry BS Scriba TJ Snowden MA Lockhart S. Safety and efficacy of MVA85A, a new tuberculosis vaccine, in infants previously vaccinated with BCG: a randomised, placebo-controlled phase 2b trial. Lancet. (2013) 381:1021–8. 10.1016/S0140-6736(13)60177-4 23391465 PMC5424647

[7] Kagina BM Abel B Scriba TJ Hughes EJ Keyser A Soares A. Specific T cell frequency and cytokine expression profile do not correlate with protection against tuberculosis after bacillus Calmette-Guerin vaccination of newborns. Am J Respir Crit Care Med. (2010) 182:1073–9. 10.1164/rccm.201003-0334OC 20558627 PMC2970848

[8] Steigler P Verrall AJ Kirman JR. Beyond memory T cells: mechanisms of protective immunity to tuberculosis infection. Immunol Cell Biol. (2019) 97:647–55. 10.1111/imcb.12278 31141205

[9] Hansen SG Zak DE Xu G Ford JC Marshall EE Malouli D. Prevention of tuberculosis in rhesus macaques by a cytomegalovirus-based vaccine. Nat Med. (2018) 24:130–43. 10.1038/nm.4473 29334373 PMC5909823

[10] Lu LL Chung AW Rosebrock TR Ghebremichael M Yu WH Grace PS. A functional role for antibodies in tuberculosis. Cell. (2016) 167:433–43.e14. 10.1016/j.cell.2016.08.072 27667685 PMC5526202

[11] Kaufmann E Sanz J Dunn JL Khan N Mendonca LE Pacis A. BCG educates hematopoietic stem cells to generate protective innate immunity against tuberculosis. Cell. (2018) 172:176–90.e19. 10.1016/j.cell.2017.12.031 29328912

[12] Ardain A Domingo-Gonzalez R Das S Kazer SW Howard NC Singh A. Group 3 innate lymphoid cells mediate early protective immunity against tuberculosis. Nature. (2019) 570:528–32. 10.1038/s41586-019-1276-2 31168092 PMC6626542

[13] Choreño Parra JA Martinez Zuniga N Jimenez Zamudio LA Jimenez Alvarez LA Salinas Lara C Zuniga J. Memory of natural killer cells: a new chance against *Mycobacterium tuberculosis*? Front Immunol. (2017) 8:967. 10.3389/fimmu.2017.00967 28855906 PMC5558047

[14] Rao M Valentini D Poiret T Dodoo E Parida S Zumla A B in TB: B cells as mediators of clinically relevant immune responses in tuberculosis. Clin Infect Dis. (2015) 61(Suppl 3):S225–34. 10.1093/cid/civ614 PMC4583574 26409285

[15] Venkatasubramanian S Cheekatla S Paidipally P Tripathi D Welch E Tvinnereim AR. IL-21-dependent expansion of memory-like NK cells enhances protective immune responses against *Mycobacterium tuberculosis*. Mucosal Immunol. (2017) 10:1031–42. 10.1038/mi.2016.105 27924822 PMC5462891

[16] Verrall AJ Alisjahbana B Apriani L Novianty N Nurani AC van Laarhoven A. Early clearance of *Mycobacterium tuberculosis*: the INFECT case contact cohort study in Indonesia. J Infect Dis. (2019). [Epub ahead of print]. 10.1093/infdis/jiz168 31298280

[17] Ewer K Millington KA Deeks JJ Alvarez L Bryant G Lalvani A. Dynamic antigen-specific T-cell responses after point-source exposure to *Mycobacterium tuberculosis*. Am J Respir Crit Care Med. (2006) 174:831–9. 10.1164/rccm.200511-1783OC 16799072

[18] Netea MG Joosten LA Latz E Mills KH Natoli G Stunnenberg HG. Trained immunity: a program of innate immune memory in health and disease. Science. (2016) 352:aaf1098. 10.1126/science.aaf1098 27102489 PMC5087274

[19] Chedid L Parant M Parant F Lefrancher P Choay J Lederer E. Enhancement of nonspecific immunity to *Klebsiella pneumoniae* infection by a synthetic immunoadjuvant (N-acetylmuramyl-L-alanyl-D-isoglutamine) and several analogs. Proc Natl Acad Sci USA. (1977) 74:2089–93. 10.1073/pnas.74.5.2089 325566 PMC431080

[20] Gorhe DS. Inhibition of multiplication of foot and mouth disease virus in adult mice pretreated with Freund's complete adjuvant. Nature. (1967) 216:1242–4. 10.1038/2161242a0 4294738

[21] van ‘t Wout JW Poell R van Furth R. The role of BCG/PPD-activated macrophages in resistance against systemic candidiasis in mice. Scand J Immunol. (1992) 36:713–9. 10.1111/j.1365-3083.1992.tb03132.x 1439583

[22] Tribouley J Tribouley-Duret J Appriou M. Effect of Bacillus Callmette Guerin (BCG) on the receptivity of nude mice to *Schistosoma mansoni*. C R Seances Soc Biol Fil. (1978) 172:902–4. 157204

[23] Hollm-Delgado MG Stuart EA Black RE. Acute lower respiratory infection among Bacille Calmette-Guerin (BCG)-vaccinated children. Pediatrics. (2014) 133:e73–81. 10.1542/peds.2013-2218 24379224

[24] de Castro MJ Pardo-Seco J Martinon-Torres F. Nonspecific (Heterologous) protection of neonatal bcg vaccination against hospitalization due to respiratory infection and sepsis. Clin Infect Dis. (2015) 60:1611–9. 10.1093/cid/civ144 25725054

[25] Arts RJW Moorlag S Novakovic B Li Y Wang SY Oosting M. BCG vaccination protects against experimental viral infection in humans through the induction of cytokines associated with trained immunity. Cell Host Microbe. (2018) 23:89–100.e5. 10.1016/j.chom.2017.12.010 29324233

[26] Elliott AM Nakiyingi J Quigley MA French N Gilks CF Whitworth JA. Inverse association between BCG immunisation and intestinal nematode infestation among HIV-1-positive individuals in Uganda. Lancet. (1999) 354:1000–1. 10.1016/S0140-6736(99)03290-0 10501367

[27] Davies LC Jenkins SJ Allen JE Taylor PR. Tissue-resident macrophages. Nat. Immunol. (2013) 14:986–95. 10.1038/ni.2705 24048120 PMC4045180

[28] Yona S Kim KW Wolf Y Mildner A Varol D Breker M. Fate mapping reveals origins and dynamics of monocytes and tissue macrophages under homeostasis. Immunity. (2013) 38:79–91. 10.1016/j.immuni.2012.12.001 23273845 PMC3908543

[29] Dunlap MD Howard N Das S Scott N Ahmed M Prince O. A novel role for C-C motif chemokine receptor 2 during infection with hypervirulent *Mycobacterium tuberculosis*. Mucosal Immunol. (2018) 11:1727–42. 10.1038/s41385-018-0071-y 30115997 PMC6279476

[30] Cai Y Sugimoto C Arainga M Alvarez X Didier ES Kuroda MJ. *In vivo* characterization of alveolar and interstitial lung macrophages in rhesus macaques: implications for understanding lung disease in humans. J Immunol. (2014) 192:2821–9. 10.4049/jimmunol.1302269 24534529 PMC3959879

[31] Tsai MC Chakravarty S Zhu G Xu J Tanaka K Koch C. Characterization of the tuberculous granuloma in murine and human lungs: cellular composition and relative tissue oxygen tension. Cell Microbiol. (2006) 8:218–32. 10.1111/j.1462-5822.2005.00612.x 16441433

[32] Zuñiga J Torres-García D Santos-Mendoza T Rodriguez-Reyna TS Granados J Yunis EJ. Cellular and humoral mechanisms involved in the control of tuberculosis. Clin Dev Immunol. (2012) 2012:193923. 10.1155/2012/193923 22666281 PMC3362816

[33] Ernst JD. Mechanisms of *M. tuberculosis* immune evasion as challenges to TB vaccine design. Cell Host Microbe. (2018) 24:34–42. 10.1016/j.chom.2018.06.004 30001523 PMC6482466

[34] Slight SR Rangel-Moreno J Gopal R Lin Y Fallert Junecko BA Mehra S. CXCR5(+) T helper cells mediate protective immunity against tuberculosis. J Clin Invest. (2013) 123:712–26. 10.1172/JCI65728 23281399 PMC3561804

[35] Sakai S Kauffman KD Schenkel JM McBerry CC Mayer-Barber KD Masopust D. Cutting edge: control of *Mycobacterium tuberculosis* infection by a subset of lung parenchyma-homing CD4 T cells. J Immunol. (2014) 192:2965–9. 10.4049/jimmunol.1400019 24591367 PMC4010124

[36] Kleinnijenhuis J Quintin J Preijers F Joosten LA Ifrim DC Saeed S. Bacille Calmette-Guerin induces NOD2-dependent nonspecific protection from reinfection via epigenetic reprogramming of monocytes. Proc Natl Acad Sci USA. (2012) 109:17537–42. 10.1073/pnas.1202870109 22988082 PMC3491454

[37] Cywes C Hoppe HC Daffe M Ehlers MR. Nonopsonic binding of *Mycobacterium tuberculosis* to complement receptor type 3 is mediated by capsular polysaccharides and is strain dependent. Infect Immun. (1997) 65:4258–66. 10.1128/IAI.65.10.4258-4266.1997 9317035 PMC175611

[38] Melo MD Catchpole IR Haggar G Stokes RW. Utilization of CD11b knockout mice to characterize the role of complement receptor 3 (CR3, CD11b/CD18) in the growth of *Mycobacterium tuberculosis* in macrophages. Cell Immunol. (2000) 205:13–23. 10.1006/cimm.2000.1710 11078603

[39] Lv J He X Wang H Wang Z Kelly GT Wang X. TLR4-NOX2 axis regulates the phagocytosis and killing of *Mycobacterium tuberculosis* by macrophages. BMC Pulm Med. (2017) 17:194. 10.1186/s12890-017-0517-0 29233104 PMC5727946

[40] Brooks MN Rajaram MV Azad AK Amer AO Valdivia-Arenas MA Park JH. NOD2 controls the nature of the inflammatory response and subsequent fate of *Mycobacterium tuberculosis* and *M. bovis* BCG in human macrophages. Cell Microbiol. (2011) 13:402–18. 10.1111/j.1462-5822.2010.01544.x 21040358 PMC3259431

[41] Homer CR Kabi A Marina-Garcia N Sreekumar A Nesvizhskii AI Nickerson KP. A dual role for receptor-interacting protein kinase 2 (RIP2) kinase activity in nucleotide-binding oligomerization domain 2 (NOD2)-dependent autophagy. J Biol Chem. (2012) 287:25565–76. 10.1074/jbc.M111.326835 22665475 PMC3408141

[42] Buffen K Oosting M Quintin J Ng A Kleinnijenhuis J Kumar V. Autophagy controls BCG-induced trained immunity and the response to intravesical BCG therapy for bladder cancer. PLoS Pathog. (2014) 10:e1004485. 10.1371/journal.ppat.1004485 25356988 PMC4214925

[43] Patel AA Zhang Y Fullerton JN Boelen L Rongvaux A Maini AA. The fate and lifespan of human monocyte subsets in steady state and systemic inflammation. J Exp Med. (2017) 214:1913–23. 10.1084/jem.20170355 28606987 PMC5502436

[44] Burgess SL Buonomo E Carey M Cowardin C Naylor C Noor Z. Bone marrow dendritic cells from mice with an altered microbiota provide interleukin 17A-dependent protection against Entamoeba histolytica colitis. MBio. (2014) 5:e01817. 10.1128/mBio.01817-14 25370489 PMC4222101

[45] Saeed S Quintin J Kerstens HH Rao NA Aghajanirefah A Matarese F. Epigenetic programming of monocyte-to-macrophage differentiation and trained innate immunity. Science. (2014) 345:1251086. 10.1126/science.1251086 25258085 PMC4242194

[46] Arts RJW Carvalho A La Rocca C Palma C Rodrigues F Silvestre R. Immunometabolic pathways in BCG-induced trained immunity. Cell Rep. (2016) 17:2562–2571. 10.1016/j.celrep.2016.11.011 27926861 PMC5177620

[47] Wendeln AC Degenhardt K Kaurani L Gertig M Ulas T Jain G. Innate immune memory in the brain shapes neurological disease hallmarks. Nature. (2018) 556:332–8. 10.1038/s41586-018-0023-4 29643512 PMC6038912

[48] Christ A Bekkering S Latz E Riksen NP. Long-term activation of the innate immune system in atherosclerosis. Semin Immunol. (2016) 28:384–93. 10.1016/j.smim.2016.04.004 27113267

[49] Groh L Keating ST Joosten LAB Netea MG Riksen NP. Monocyte and macrophage immunometabolism in atherosclerosis. Semin Immunopathol. (2018) 40:203–14. 10.1007/s00281-017-0656-7 28971272 PMC5809534

[50] Darrah PA Zeppa JJ Maiello P Hackney JA Wadsworth MH 2nd Hughes TK. Prevention of tuberculosis in macaques after intravenous BCG immunization. Nature. (2020) 577:95–102. 10.1038/s41586-019-1817-8 31894150 PMC7015856

[51] Yao Y Jeyanathan M Haddadi S Barra NG Vaseghi-Shanjani M Damjanovic D. Induction of autonomous memory alveolar macrophages requires T cell help and is critical to trained immunity. Cell. (2018) 175:1634–50.e17. 10.1016/j.cell.2018.09.042 30433869

[52] Chen L Wang J Zganiacz A Xing Z. Single intranasal mucosal Mycobacterium bovis BCG vaccination confers improved protection compared to subcutaneous vaccination against pulmonary tuberculosis. Infect Immun. (2004) 72:238–46. 10.1128/IAI.72.1.238-246.2004 14688101 PMC344011

[53] Perdomo C Zedler U Kuhl AA Lozza L Saikali P Sander LE. Mucosal BCG vaccination induces protective lung-resident memory T Cell populations against tuberculosis. MBio. (2016) 7:e01686–16. 10.1128/mBio.01686-16 27879332 PMC5120139

[54] Dijkman K Sombroek CC Vervenne RAW Hofman SO Boot C Remarque EJ. Prevention of tuberculosis infection and disease by local BCG in repeatedly exposed rhesus macaques. Nat Med. (2019) 25:255–62. 10.1038/s41591-018-0319-9 30664782

[55] Huang L Nazarova EV Tan S Liu Y Russell DG. Growth of *Mycobacterium tuberculosis in vivo* segregates with host macrophage metabolism and ontogeny. J Exp Med. (2018) 215:1135–52. 10.1084/jem.20172020 29500179 PMC5881470

[56] Lodoen MB Lanier LL. Natural killer cells as an initial defense against pathogens. Curr Opin Immunol. (2006) 18:391–8. 10.1016/j.coi.2006.05.002 16765573 PMC7127478

[57] Sivori S Falco M Della Chiesa M Carlomagno S Vitale M Moretta L. CpG and double-stranded RNA trigger human NK cells by Toll-like receptors: induction of cytokine release and cytotoxicity against tumors and dendritic cells. Proc Natl Acad Sci USA. (2004) 101:10116–21. 10.1073/pnas.0403744101 15218108 PMC454174

[58] Marcenaro E Ferranti B Falco M Moretta L Moretta A. Human NK cells directly recognize Mycobacterium bovis via TLR2 and acquire the ability to kill monocyte-derived DC. Int Immunol. (2008) 20:1155–67. 10.1093/intimm/dxn073 18596023

[59] Long EO Kim HS Liu D Peterson ME Rajagopalan S. Controlling natural killer cell responses: integration of signals for activation and inhibition. Annu Rev Immunol. (2013) 31:227–58. 10.1146/annurev-immunol-020711-075005 23516982 PMC3868343

[60] Lanier LL. Up on the tightrope: natural killer cell activation and inhibition. Nat Immunol. (2008) 9:495–502. 10.1038/ni1581 18425106 PMC2669298

[61] Orange JS Wang B Terhorst C Biron CA. Requirement for natural killer cell-produced interferon gamma in defense against murine cytomegalovirus infection and enhancement of this defense pathway by interleukin 12 administration. J Exp Med. (1995) 182:1045–56. 10.1084/jem.182.4.1045 7561678 PMC2192290

[62] Wang R Jaw JJ Stutzman NC Zou Z Sun PD. Natural killer cell-produced IFN-gamma and TNF-alpha induce target cell cytolysis through up-regulation of ICAM-1. J Leukoc Biol. (2012) 91:299–309. 10.1189/jlb.0611308 22045868 PMC3290424

[63] Passos ST Silver JS O'Hara AC Sehy D Stumhofer JS Hunter CA. IL-6 promotes NK cell production of IL-17 during toxoplasmosis. J Immunol. (2010) 184:1776–83. 10.4049/jimmunol.0901843 20083665 PMC3757499

[64] Kumar P Thakar MS Ouyang W Malarkannan S. IL-22 from conventional NK cells is epithelial regenerative and inflammation protective during influenza infection. Mucosal Immunol. (2013) 6:69–82. 10.1038/mi.2012.49 22739232 PMC3835350

[65] Xu X Weiss ID Zhang HH Singh SP Wynn TA Wilson MS. Conventional NK cells can produce IL-22 and promote host defense in *Klebsiella pneumoniae* pneumonia. J Immunol. (2014) 192:1778–86. 10.4049/jimmunol.1300039 24442439 PMC3995347

[66] Cuturi MC Anegon I Sherman F Loudon R Clark SC Perussia B. Production of hematopoietic colony-stimulating factors by human natural killer cells. J Exp Med. (1989) 169:569–83. 10.1084/jem.169.2.569 2521357 PMC2189209

[67] Domingo-Gonzalez R Prince O Cooper A Khader SA. Cytokines and chemokines in *Mycobacterium tuberculosis* infection. Microbiol Spectr. (2016) 4:TBTB2-0018-2016. 10.1128/microbiolspec.TBTB2-0018-2016 27763255 PMC5205539

[68] Esin S Batoni G Counoupas C Stringaro A Brancatisano FL Colone M. Direct binding of human NK cell natural cytotoxicity receptor NKp44 to the surfaces of mycobacteria and other bacteria. Infect Immun. (2008) 76:1719–27. 10.1128/IAI.00870-07 18212080 PMC2292874

[69] Esin S Counoupas C Aulicino A Brancatisano FL Maisetta G Bottai D. Interaction of *Mycobacterium tuberculosis* cell wall components with the human natural killer cell receptors NKp44 and Toll-like receptor 2. Scand J Immunol. (2013) 77:460–9. 10.1111/sji.12052 23578092

[70] Dhiman R Indramohan M Barnes PF Nayak RC Paidipally P Rao LV. IL-22 produced by human NK cells inhibits growth of *Mycobacterium tuberculosis* by enhancing phagolysosomal fusion. J Immunol. (2009) 183:6639–45. 10.4049/jimmunol.0902587 19864591

[71] Guerra C Johal K Morris D Moreno S Alvarado O Gray D. Control of *Mycobacterium tuberculosis* growth by activated natural killer cells. Clin Exp Immunol. (2012) 168:142–52. 10.1111/j.1365-2249.2011.04552.x 22385249 PMC3390505

[72] Dhiman R Venkatasubramanian S Paidipally P Barnes PF Tvinnereim A Vankayalapati R. Interleukin 22 inhibits intracellular growth of *Mycobacterium tuberculosis* by enhancing calgranulin A expression. J Infect Dis. (2014) 209:578–87. 10.1093/infdis/jit495 24041785 PMC3903372

[73] Vankayalapati R Garg A Porgador A Griffith DE Klucar P Safi H. Role of NK cell-activating receptors and their ligands in the lysis of mononuclear phagocytes infected with an intracellular bacterium. J Immunol. (2005) 175:4611–7. 10.4049/jimmunol.175.7.4611 16177106

[74] Lu CC Wu TS Hsu YJ Chang CJ Lin CS Chia JH. NK cells kill mycobacteria directly by releasing perforin and granulysin. J Leukoc Biol. (2014) 96:1119–29. 10.1189/jlb.4A0713-363RR 25139289

[75] Feng CG Kaviratne M Rothfuchs AG Cheever A Hieny S Young HA. NK cell-derived IFN-gamma differentially regulates innate resistance and neutrophil response in T cell-deficient hosts infected with *Mycobacterium tuberculosis*. J Immunol. (2006) 177:7086–93. 10.4049/jimmunol.177.10.7086 17082625

[76] Paidipally P Tripathi D Van A Radhakrishnan RK Dhiman R Venkatasubramanian S. Interleukin-21 regulates natural killer cell responses during *Mycobacterium tuberculosis* infection. J Infect Dis. (2018) 217:1323–33. 10.1093/infdis/jiy034 29390153 PMC6018723

[77] Junqueira-Kipnis AP Kipnis A Jamieson A Juarrero MG Diefenbach A Raulet DH. NK cells respond to pulmonary infection with *Mycobacterium tuberculosis*, but play a minimal role in protection. J Immunol. (2003) 171:6039–45. 10.4049/jimmunol.171.11.6039 14634116

[78] Portevin D Via LE Eum S Young D. Natural killer cells are recruited during pulmonary tuberculosis and their ex vivo responses to mycobacteria vary between healthy human donors in association with KIR haplotype. Cell Microbiol. (2012) 14:1734–44. 10.1111/j.1462-5822.2012.01834.x 22788220 PMC3503254

[79] Mendez A Granda H Meenagh A Contreras S Zavaleta R Mendoza MF. Study of KIR genes in tuberculosis patients. Tissue Antigens. (2006) 68:386–9. 10.1111/j.1399-0039.2006.00685.x 17092251

[80] Mahfouz R Halas H Hoteit R Saadeh M Shamseddeen W Charafeddine K. Study of KIR genes in Lebanese patients with tuberculosis. Int J Tuberc Lung Dis. (2011) 15:1688–91. 10.5588/ijtld.11.0138 22118180

[81] Pydi SS Sunder SR Venkatasubramanian S Kovvali S Jonnalagada S Valluri VL. Killer cell immunoglobulin like receptor gene association with tuberculosis. Hum Immunol. (2013) 74:85–92. 10.1016/j.humimm.2012.10.006 23073291

[82] Salie M Daya M Moller M Hoal EG. Activating KIRs alter susceptibility to pulmonary tuberculosis in a South African population. Tuberculosis. (2015) 95:817–21. 10.1016/j.tube.2015.09.003 26542219

[83] Batoni G Esin S Favilli F Pardini M Bottai D Maisetta G. Human CD56bright and CD56dim natural killer cell subsets respond differentially to direct stimulation with *Mycobacterium bovis* bacillus Calmette-Guerin. Scand J Immunol. (2005) 62:498–506. 10.1111/j.1365-3083.2005.01692.x 16316416

[84] Bozzano F Costa P Passalacqua G Dodi F Ravera S Pagano G. Functionally relevant decreases in activatory receptor expression on NK cells are associated with pulmonary tuberculosis *in vivo* and persist after successful treatment. Int Immunol. (2009) 21:779–91. 10.1093/intimm/dxp046 19461127

[85] Barcelos W Sathler-Avelar R Martins-Filho OA Carvalho BN Guimaraes TM Miranda SS. Natural killer cell subpopulations in putative resistant individuals and patients with active *Mycobacterium tuberculosis* infection. Scand J Immunol. (2008) 68:92–102. 10.1111/j.1365-3083.2008.02116.x 18484953

[86] Fan R Xiang Y Yang L Liu Y Chen P Wang L. Impaired NK cells' activity and increased numbers of CD4 + CD25+ regulatory T cells in multidrug-resistant *Mycobacterium tuberculosis* patients. Tuberculosis. (2016) 98:13–20. 10.1016/j.tube.2016.02.001 27156613

[87] Garand M Goodier M Owolabi O Donkor S Kampmann B Sutherland JS. Functional and phenotypic changes of natural killer cells in whole blood during *Mycobacterium tuberculosis* infection and disease. Front Immunol. (2018) 9:257. 10.3389/fimmu.2018.00257 29520269 PMC5827559

[88] Roy Chowdhury R Vallania F Yang Q Lopez Angel CJ Darboe F Penn-Nicholson A. A multi-cohort study of the immune factors associated with *M. tuberculosis* infection outcomes. Nature. (2018) 560:644–8. 10.1038/s41586-018-0439-x 30135583 PMC6414221

[89] Vankayalapati R Klucar P Wizel B Weis SE Samten B Safi H. NK cells regulate CD8+ T cell effector function in response to an intracellular pathogen. J Immunol. (2004) 172:130–7. 10.4049/jimmunol.172.1.130 14688318

[90] Ferlazzo G Tsang ML Moretta L Melioli G Steinman RM Munz C. Human dendritic cells activate resting natural killer (NK) cells and are recognized via the NKp30 receptor by activated NK cells. J Exp Med. (2002) 195:343–51. 10.1084/jem.20011149 11828009 PMC2193591

[91] Roy S Barnes PF Garg A Wu S Cosman D Vankayalapati R. NK cells lyse T regulatory cells that expand in response to an intracellular pathogen. J Immunol. (2008) 180:1729–36. 10.4049/jimmunol.180.3.1729 18209070

[92] Dhiman R Periasamy S Barnes PF Jaiswal AG Paidipally P Barnes AB. NK1.1+ cells and IL-22 regulate vaccine-induced protective immunity against challenge with Mycobacterium tuberculosis. J Immunol. (2012) 189:897–905. 10.4049/jimmunol.1102833 22711885 PMC3392427

[93] O'Leary JG Goodarzi M Drayton DL von Andrian UH. T cell- and B cell-independent adaptive immunity mediated by natural killer cells. Nat Immunol. (2006) 7:507–16. 10.1038/ni1332 16617337

[94] Sun JC Beilke JN Lanier LL. Adaptive immune features of natural killer cells. Nature. (2009) 457:557–61. 10.1038/nature07665 19136945 PMC2674434

[95] Habib S El Andaloussi A Hisham A Ismail N. NK cell-mediated regulation of protective memory responses against intracellular ehrlichial pathogens. PLoS ONE. (2016) 11:e0153223. 10.1371/journal.pone.0153223 27092553 PMC4836677

[96] Foley B Cooley S Verneris MR Curtsinger J Luo X Waller EK. Human cytomegalovirus (CMV)-induced memory-like NKG2C(+) NK cells are transplantable and expand *in vivo* in response to recipient CMV antigen. J Immunol. (2012) 189:5082–8. 10.4049/jimmunol.1201964 23077239 PMC3490031

[97] Cooper MA Elliott JM Keyel PA Yang L Carrero JA Yokoyama WM. Cytokine-induced memory-like natural killer cells. Proc Natl Acad Sci USA. (2009) 106:1915–9. 10.1073/pnas.0813192106 19181844 PMC2644138

[98] Romee R Schneider SE Leong JW Chase JM Keppel CR Sullivan RP. Cytokine activation induces human memory-like NK cells. Blood. (2012) 120:4751–60. 10.1182/blood-2012-04-419283 22983442 PMC3520618

[99] Kleinnijenhuis J Quintin J Preijers F Joosten LA Jacobs C Xavier RJ. BCG-induced trained immunity in NK cells: role for non-specific protection to infection. Clin Immunol. (2014) 155:213–9. 10.1016/j.clim.2014.10.005 25451159 PMC5084088

[100] Suliman S Geldenhuys H Johnson JL Hughes JE Smit E Murphy M. Bacillus Calmette-Guerin (BCG) revaccination of adults with latent *Mycobacterium tuberculosis* infection induces long-lived BCG-reactive NK cell responses. J Immunol. (2016) 197:1100–10. 10.4049/jimmunol.1501996 27412415 PMC4976036

[101] Fu X Yu S Yang B Lao S Li B Wu C. Memory-like antigen-specific human NK cells from TB pleural fluids produced IL-22 in response to IL-15 or *Mycobacterium tuberculosis* antigens. PLoS ONE. (2016) 11:e0151721. 10.1371/journal.pone.0151721 27031950 PMC4816314

[102] Fu X Liu Y Li L Li Q Qiao D Wang H. Human natural killer cells expressing the memory-associated marker CD45RO from tuberculous pleurisy respond more strongly and rapidly than CD45RO- natural killer cells following stimulation with interleukin-12. Immunology. (2011) 134:41–9. 10.1111/j.1365-2567.2011.03464.x 21711347 PMC3173693

[103] Diefenbach A Colonna M Koyasu S. Development, differentiation, and diversity of innate lymphoid cells. Immunity. (2014) 41:354–65. 10.1016/j.immuni.2014.09.005 25238093 PMC4171710

[104] Burkhardt AM Maravillas-Montero JL Carnevale CD Vilches-Cisneros N Flores JP Hevezi PA. CXCL17 is a major chemotactic factor for lung macrophages. J Immunol. (2014) 193:1468–74. 10.4049/jimmunol.1400551 24973458 PMC4142799

[105] Tripathi D Radhakrishnan RK Sivangala Thandi R Paidipally P Devalraju KP Neela VSK. IL-22 produced by type 3 innate lymphoid cells (ILC3s) reduces the mortality of type 2 diabetes mellitus (T2DM) mice infected with *Mycobacterium tuberculosis*. PLoS Pathog. (2019) 15:e1008140. 10.1371/journal.ppat.1008140 31809521 PMC6919622

[106] Wang X Peng H Tian Z. Innate lymphoid cell memory. Cell Mol Immunol. (2019) 16:423–9. 10.1038/s41423-019-0212-6 30796350 PMC6474199

[107] Steigler P Daniels NJ McCulloch TR Ryder BM Sandford SK Kirman JR. BCG vaccination drives accumulation and effector function of innate lymphoid cells in murine lungs. Immunol Cell Biol. (2018) 96:379–89. 10.1111/imcb.12007 29363172

[108] Neuberger MS Ehrenstein MR Rada C Sale J Batista FD Williams G. Memory in the B-cell compartment: antibody affinity maturation. Philos Trans R Soc Lond B Biol Sci. (2000) 355:357–60. 10.1098/rstb.2000.0573 10794054 PMC1692737

[109] De Silva NS Klein U. Dynamics of B cells in germinal centres. Nat Rev Immunol. (2015) 15:137–48. 10.1038/nri3804 25656706 PMC4399774

[110] Glatman-Freedman A Casadevall A. Serum therapy for tuberculosis revisited: reappraisal of the role of antibody-mediated immunity against *Mycobacterium tuberculosis*. Clin Microbiol Rev. (1998) 11:514–32. 10.1128/CMR.11.3.514 9665981 PMC88894

[111] Encinales L Zuniga J Granados-Montiel J Yunis M Granados J Almeciga I. Humoral immunity in tuberculin skin test anergy and its role in high-risk persons exposed to active tuberculosis. Mol Immunol. (2010) 47:1066–73. 10.1016/j.molimm.2009.11.005 20004475 PMC2815102

[112] Lu LL Smith MT Yu KKQ Luedemann C Suscovich TJ Grace PS. IFN-γ-independent immune markers of *Mycobacterium tuberculosis* exposure. Nat Med. (2019) 25:977–987. 10.1038/s41591-019-0441-3 31110348 PMC6559862

[113] Li H Javid B. Antibodies and tuberculosis: finally coming of age? Nat Rev Immunol. (2018) 18:591–596. 10.1038/s41577-018-0028-0 29872140

[114] Simmons JD Stein CM Seshadri C Campo M Alter G Fortune S. Immunological mechanisms of human resistance to persistent *Mycobacterium tuberculosis* infection. Nat Rev Immunol. (2018) 18:575–589. 10.1038/s41577-018-0025-3 29895826 PMC6278832

[115] du Plessis WJ Kleynhans L du Plessis N Stanley K Malherbe ST Maasdorp E. The functional response of B cells to antigenic stimulation: a preliminary report of latent tuberculosis. PLoS ONE. (2016) 11:e0152710. 10.1371/journal.pone.0152710 27050308 PMC4822853

[116] du Plessis WJ Keyser A Walzl G Loxton AG. Phenotypic analysis of peripheral B cell populations during *Mycobacterium tuberculosis* infection and disease. J Inflamm. (2016) 13:23. 10.1186/s12950-016-0133-4 27478412 PMC4966581

[117] du Plessis WJ Walzl G Loxton AG. B cells as multi-functional players during *Mycobacterium tuberculosis* infection and disease. Tuberculosis. (2016) 97:118–25. 10.1016/j.tube.2015.10.007 26611659

[118] Sebina I Biraro IA Dockrell HM Elliott AM Cose S. Circulating B-lymphocytes as potential biomarkers of tuberculosis infection activity. PLoS ONE. (2014) 9:e106796. 10.1371/journal.pone.0106796 25192196 PMC4156407

[119] Zimmermann N Thormann V Hu B Köhler AB Imai-Matsushima A Locht C. Human isotype-dependent inhibitory antibody responses against *Mycobacterium tuberculosis*. EMBO Mol Med. (2016) 8:1325–39. 10.15252/emmm.201606330 27729388 PMC5090662

[120] Abreu MT Carvalheiro H Rodrigues-Sousa T Domingos A Segorbe-Luis A Rodrigues-Santos P. Alterations in the peripheral blood B cell subpopulations of multidrug-resistant tuberculosis patients. Clin Exp Med. (2014) 14:423–9. 10.1007/s10238-013-0258-1 24068613

[121] Schierloh P Landoni V Balboa L Musella RM Castagnino J Morana E. Human pleural B-cells regulate IFN-gamma production by local T-cells and NK cells in a *Mycobacterium tuberculosis*-induced delayed hypersensitivity reaction. Clin Sci. (2014) 127:391–403. 10.1042/CS20130769 24689690

[122] Sebina I Cliff JM Smith SG Nogaro S Webb EL Riley EM. Long-lived memory B-cell responses following BCG vaccination. PLoS ONE. (2012) 7:e51381. 10.1371/journal.pone.0051381 23240017 PMC3519837

[123] Maglione PJ Xu J Chan J. B cells moderate inflammatory progression and enhance bacterial containment upon pulmonary challenge with *Mycobacterium tuberculosis*. J Immunol. (2007) 178:7222–34. 10.4049/jimmunol.178.11.7222 17513771

[124] Chan J Mehta S Bharrhan S Chen Y Achkar JM Casadevall A. The role of B cells and humoral immunity in *Mycobacterium tuberculosis* infection. Semin Immunol. (2014) 26:588–600. 10.1016/j.smim.2014.10.005 25458990 PMC4314354

[125] Loxton AG. B cells and their regulatory functions during tuberculosis: latency and active disease. Mol Immunol. (2019) 111:145–51. 10.1016/j.molimm.2019.04.012 31054408

[126] HayGlass KT Naides SJ Benacerraf B Sy MS. T cell development in B cell deficient mice. III. Restriction specificity of suppressor T cell factor(s) produced in mice treated chronically with rabbit anti-mouse mu chain antibody. J Mol Cell Immunol. (1985) 2:107–17. 2978459

[127] van Rensburg IC Wagman C Stanley K Beltran C Ronacher K Walzl G. Successful TB treatment induces B-cells expressing FASL and IL5RA mRNA. Oncotarget. (2017) 8:2037–43. 10.18632/oncotarget.12184 27682872 PMC5356777

[128] Joosten SA van Meijgaarden KE Del Nonno F Baiocchini A Petrone L Vanini V. Patients with tuberculosis have a dysfunctional circulating B-cell compartment, which normalizes following successful treatment. PLoS Pathog. (2016) 12:e1005687. 10.1371/journal.ppat.1005687 27304615 PMC4909319

[129] Phuah J Wong EA Gideon HP Maiello P Coleman MT Hendricks MR. Effects of B cell depletion on Early *Mycobacterium tuberculosis* infection in cynomolgus macaques. Infect Immun. (2016) 84:1301–11. 10.1128/IAI.00083-16 26883591 PMC4862708

[130] Ulrichs T Kosmiadi GA Trusov V Jorg S Pradl L Titukhina M. Human tuberculous granulomas induce peripheral lymphoid follicle-like structures to orchestrate local host defence in the lung. J Pathol. (2004) 204:217–28. 10.1002/path.1628 15376257

[131] Sable SB Posey JE Scriba TJ. Tuberculosis vaccine development: progress in clinical evaluation. Clin Microbiol Rev. (2019) 33:e00100–19. 10.1128/CMR.00100-19 31666281 PMC6822991

[132] Almeida LP Trombone AP Lorenzi JC Rocha CD Malardo T Fontoura IC. B cells can modulate the CD8 memory T cell after DNA vaccination against experimental tuberculosis. Genet Vaccines Ther. (2011) 9:5. 10.1186/1479-0556-9-5 21401938 PMC3066104

[133] Khader SA Divangahi M Hanekom W Hill PC Maeurer M Makar KW. Targeting innate immunity for tuberculosis vaccination. J Clin Invest. (2019) 129:3482–91. 10.1172/JCI128877 31478909 PMC6715374

